# Optic Neuritis in a Child With COVID-19: A Rare Association

**DOI:** 10.7759/cureus.14094

**Published:** 2021-03-24

**Authors:** Yusuf Parvez, Fatma AlZarooni, Farheen Khan

**Affiliations:** 1 Pediatric Medicine, Dubai Hospital, Dubai, ARE; 2 Pediatrics and Child Health, Latifa Women's and Children's Hospital, Dubai, ARE; 3 Pediatrics, Dubai Hospital, Dubai, ARE

**Keywords:** covid-19, optic neuritis, severe acute respiratory distress syndrome

## Abstract

The recent coronavirus disease-2019 (COVID-19) pandemic has caused significant mortality and morbidity, affecting patients of all ages. COVID-19 affects various tissues and systems in the body, including the central and peripheral nervous systems. However, COVID-19 has rarely affected the eyes and caused optic neuritis. We report a unique case of COVID-19-related unilateral optic neuritis in a 10-year-old girl.

## Introduction

Coronaviruses are a group of ribonucleic acid viruses that affect birds and humans [1]. They were first discovered in the 1930s when acute respiratory tract infections were reported in domestic chickens [2]. The human coronavirus was discovered in 1960 in the United Kingdom when a boy was infected and had common cold-like symptoms [3]. Since its discovery, it has caused mild to severe infections in humans that mainly involve the respiratory tract, resulting in diseases such as severe acute respiratory syndrome, Middle East respiratory syndrome, and the recent novel coronavirus disease-2019 (COVID-19) pandemic caused by the severe acute respiratory syndrome-coronavirus-2 [4]. COVID-19 has caused significant mortality and morbidity and affects multiple organs. However, ocular manifestations have rarely been reported. We present a case of optic neuritis as a rare manifestation of COVID-19.

## Case presentation

**Figure 1:**
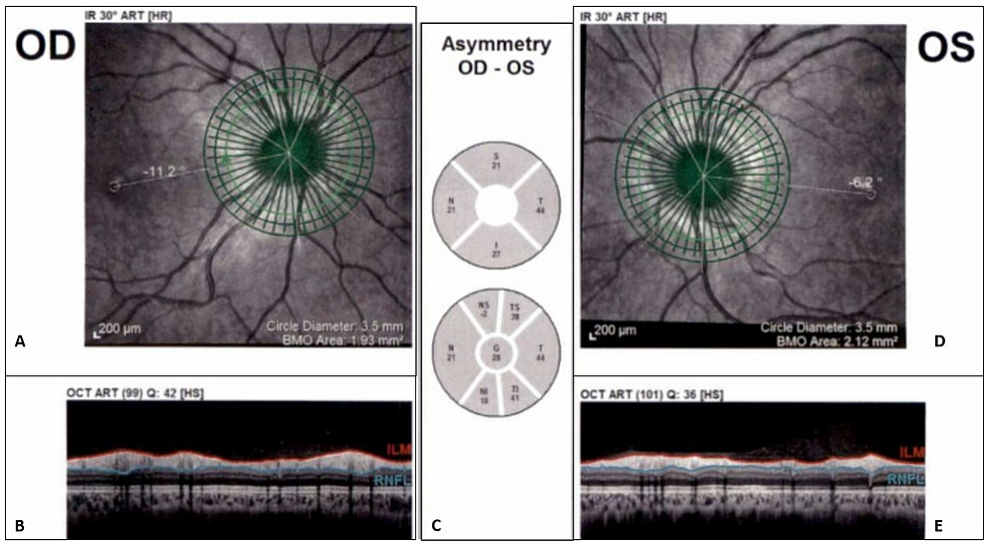
Laser tomography. (A) Right eye circle scan. (B) Right OCT scan with automatic segmentation of RNFL. (C) Differences between average thickness in sectors (along the calculation circle) in each eye. (D) Left eye circle scan. (E) Left OCT scan with automatic segmentation of RNFL. Abbreviations: ART, automatic real-time; BMO, Bruch’s membrane opening; HR, high resolution; HS, high speed; ILM, internal limiting membrane; IR, infrared reflectance imaging; OD, oculus dexter; OS, oculus sinister; OCT, optical coherence tomography; RNFL, retinal nerve fiber layer.

A 10-year-old previously healthy girl was admitted to our hospital with loss of vision in her left eye lasting for two days. She had no history of fever, pain in the eyes, vomiting, headache, seizures, or trauma. Her neurological examination results were normal, except her visual acuity was severely impaired in her left eye and normal in her right eye. She had no focal neurological deficit, and findings from her systemic examination were unremarkable. She was evaluated by a pediatric ophthalmologist, who indicated the patient had optic neuritis of the left eye (Figures 1A-1E). A pediatric neurologist recommended a magnetic resonance imaging (MRI) scan of her brain, spine, and orbit. The orbit's MRI revealed mild enlargement and slight T2 hyperintensity of the intracanalicular and intraorbital segment of the left optic nerve, confirming the diagnosis of left optic neuritis. The MRI of the brain and spine were unremarkable. The patient was admitted after collecting a COVID-19 polymerase chain reaction (PCR) swab test from her nasopharynx. She was screened for autoimmune optic neuritis, but the results from all investigations were unremarkable, including screens for anti-neuromyelitis optica, anti-myelin oligodendrocyte glycoprotein antibodies, aquaporin-4 antibodies, and myelin-associated glycoprotein antibodies. Her basic metabolic and immunologic work-ups and her cerebrospinal fluid studies revealed nothing remarkable. Her inflammatory markers, including C-reactive protein, procalcitonin, D-dimer, ferritin, and lactate dehydrogenase, were within reference limits.

Her COVID-19 PCR screen was positive, and she started intravenous pulsed methylprednisolone followed with oral prednisolone. Her vision improved after three days of hospital stay, and she was discharged on a tapering dose of prednisolone with home isolation for 14 days per the national guidelines. She was advised to follow-up in ophthalmology and pediatric clinics.

## Discussion

COVID-19 has mainly affected the respiratory system leading to life-threatening complications causing significant mortality and morbidity. However, gastrointestinal, renal, cardiac, neurological, and ocular complications have also been described in the literature [5].

The ophthalmic mode of transmission of the coronavirus has not been well studied. In 2004, human coronavirus NL63 was detected in a seven-month-old child who presented with bronchiolitis and conjunctivitis. The virus was found in tear samples of many patients [6]. Interestingly, feline coronavirus (which affects cats) and murine coronavirus (which affects mice) have caused ocular complications in humans probably because of underlying vasculitis [7,8].

COVID-19 is caused by a beta-coronavirus that uses the angiotensin-converting enzyme-2 receptor (ACE2) to enter cells. The ACE2 receptor is widely expressed in the central nervous system, including the retina and its vessels [9-11]. The ocular vascular microangiopathy in COVID-19 patients could be due to a hypercoagulable state or vasculitis, but further studies are warranted.

Our case was extensively investigated, and all other probable causes, including infection and immune-mediated etiologies of optic neuritis, were ruled out. Agrawal et al. reported that steroids are the mainstay of noninfectious uveitis treatment [12], although the treatment of optic neuritis in children due to COVID-19 has not been described in the literature. The World Health Organization declared a public health emergency of international concern on January 30, 2020, and recommended using goggles and a face shield to prevent the ocular transmission of the virus [13,14]. Additional studies are warranted to determine the etiopathogenesis and management of ocular manifestations of COVID-19.

## Conclusions

COVID-19 is a multisystem disorder. Ocular manifestations, such as conjunctivitis and optic neuritis, are rare but should not be ignored. Physicians should consider a detailed evaluation of patients using fundoscopy and MRI of the orbit when necessary. Steroids are the mainstay of treatment, but additional studies are recommended to explore the disease and successful treatments.
